# Short-Term Machine-Learning Calibration of PID Sensors for Ambient VOC OH Reactivity

**DOI:** 10.3390/s26051428

**Published:** 2026-02-25

**Authors:** Han Yang, Wei Song, Xiaoyang Wang, Jianlin Cheng, Chenglei Pei, Duohong Chen, Zhuoyue Ren, Xinyi Li, Xiangyu Zhang, Xiaodie Pang, Xue Yu, Jianqiang Zeng, Yanli Zhang, Xinming Wang

**Affiliations:** 1 State Key Laboratory of Advanced Environmental Technology, Guangzhou Institute of Geochemistry, Chinese Academy of Sciences, Guangzhou 510640, China; yanghan@gig.ac.cn (H.Y.);; 2 Guangdong Key Laboratory of Environmental Protection and Resources Utilization, Guangzhou Institute of Geochemistry, Chinese Academy of Sciences, Guangzhou 510640, China; 3 College of Resources and Environment, University of Chinese Academy of Sciences, Beijing 100049, China; 4 Guangzhou Sub-Branch of Guangdong Ecological and Environmental Monitoring Center, Guangzhou 510006, China; 5 Guangdong Ecological Environment Monitoring Center, Environmental Key Laboratory of Regional Air Quality Monitoring, Ministry of Ecology and Environment, Guangzhou 510308, China

**Keywords:** PID sensors, VOCs, machine learning, calibration, Proton Transfer Reaction Time-of-Flight Mass Spectrometer (PTR-ToF-MS), OH reactivity

## Abstract

Photoionization detector (PID) sensors are widely used for ambient Volatile organic compound (VOC) monitoring because they are inexpensive, flexible, and fast. However, PID outputs are strongly influenced by environmental conditions (especially temperature and relative humidity) and exhibit substantial inter-sensor variability, limiting their quantitative reliability. Here we present a rapid machine-learning calibration workflow that maps PID signals and meteorological covariates to a photochemically relevant reference metric, PTR-derived VOC OH reactivity (*R*_OH,PTR_, s^−1^), calculated from online PTR-ToF-MS VOC measurements weighted by OH reaction rate constants. Four MiniPID sensors were co-located with a PTR-ToF-MS and a thermohygrometer, and data were harmonized to 10-s resolution. Multiple regression models were evaluated, with ensemble methods (RF and XGBoost) providing the best overall performance. To ensure realistic generalization under temporal autocorrelation, validation used a time-aware split: models were trained on a contiguous 24-h co-location period and evaluated on subsequent days (out-of-time). In this out-of-time evaluation, XGBoost achieved strong agreement with *R*_OH,PTR_ across sensors (Pearson’s *r* = 0.85, R^2^ = 0.64, RMSE = 1.74 s^−1^), while substantially improving inter-sensor consistency. This short-duration calibration approach supports practical co-location-based harmonization of PID networks for high-temporal-resolution VOC reactivity monitoring in urban and industrial environments.

## 1. Introduction

VOCs play a central role in atmospheric chemistry and air quality. Through oxidation processes, VOCs contribute to the formation of ground-level ozone and secondary organic aerosol, and many VOC species are also directly associated with adverse health outcomes, particularly in industrial and urban environments where emissions can include numerous hazardous air pollutants [1,2,3]. Capturing VOC dynamics with high temporal and spatial resolution is therefore important for source identification, mitigation assessment, and understanding photochemical pollution episodes. Conventional online instrumentation (e.g., GC–MS, GC–FID, DOAS, FTIR) provides quantitative and/or speciated VOC measurements but is often expensive, operationally complex, and challenging to deploy at scale, limiting its suitability for dense monitoring networks [4]. These constraints motivate the development of flexible, lower-cost measurement approaches that can support distributed observations.

Low-cost sensor (LCS) systems offer advantages including compact form factor, low power requirements, and the ability to stream data in near real time, enabling deployment in spatially resolved networks [5]. For VOC monitoring, commonly used LCS technologies include electrochemical sensors, metal oxide sensors, optical sensors, and PID sensors [6]. However, electrochemical and metal oxide sensors can suffer from substantial cross-sensitivities [7], while optical approaches may remain constrained by cost and instrument complexity for certain applications [8]. PID sensors are widely used for total VOC (TVOC) monitoring due to their high sensitivity, rapid response, and low detection limits, and have been applied in diverse contexts such as biogenic emissions screening, indoor air quality assessment, marine hydrocarbon leak detection, and industrial monitoring [9,10,11,12,13].

Despite these advantages, quantitative application of PID sensors in ambient air remains challenging. PID outputs can be strongly influenced by temperature and relative humidity, and can drift with sensor aging (e.g., UV lamp intensity decay and window contamination), leading to reduced agreement with reference-grade measurements and pronounced inter-sensor variability [10,14,15,16,17,18]. In addition, standard factory calibration (often based on isobutylene) is not necessarily representative of complex real-world VOC mixtures, resulting in systematic bias when PID readings are interpreted as concentration equivalents [19]. These issues motivate the use of field calibration strategies that explicitly incorporate environmental covariates and can accommodate nonlinear sensor behavior.

Commercially available PID sensors currently in use remain inherently non-selective [20,21], with the measured signal reflecting a combined response to all VOCs whose ionization potentials fall below the lamp energy threshold. Although recent advances in sensor materials have demonstrated the potential to achieve selective detection under tailored conditions [22,23], these remain isolated exceptions and have not altered the fundamentally non-selective nature of mainstream PID sensors. This introduces a “many-to-one” calibration problem in which different VOC mixtures can produce similar PID outputs, while the same total concentration (e.g., a simple sum of VOC mixing ratios) may correspond to different PID responses depending on composition [20]. Existing calibration strategies include single- or multi-point calibration with standard gases [13], multivariate regression incorporating meteorological variables [21], and machine learning (ML) models that capture nonlinear dependencies and interactions [24]. For PID sensors in ambient air, however, calibration is additionally constrained by practical realities: sensor performance can degrade over time and under polluted conditions, making long co-location campaigns operationally costly and sometimes infeasible. Consequently, calibration approaches that can deliver robust performance from short co-location periods are of high practical value for PID network operation.

In this work, we address the PID many-to-one challenge by calibrating PID signals to a photochemically relevant integrated metric rather than to concentration alone. Specifically, we use PTR-derived VOC OH reactivity as the reference target, calculated from online VOC measurements and their reaction rate constants with the hydroxyl radical (OH). OH reactivity provides a direct measure of the effective chemical sink of OH and is commonly expressed as the sum of species-specific contributions, *R*_OH,PTR_ = Σ_*i*_ *k*_OH,*i*_[*X_i_*] [25,26]. For a non-speciated sensor such as a PID, this reactivity-weighted formulation is advantageous because (i) PID sensitivity factors can vary substantially across VOC classes, weakening the relationship between raw PID output and a simple concentration sum, whereas OH reactivity weights VOCs by *k*_OH_, elevating compounds that dominate photochemical consumption; and (ii) the VOC classes that often contribute strongly to OH reactivity in polluted environments (notably alkenes and aromatics) are generally within the sensitivity range of both PID sensors and proton-transfer-reaction mass spectrometry, providing an operationally consistent basis for constructing a reference target. In addition to improving interpretability for air quality applications, a reactivity-based target links the calibrated PID signal more directly to VOC-driven ozone formation potential and secondary pollutant production.

Accordingly, we present a short-term ML calibration workflow that maps PID raw outputs and meteorological covariates to PTR-derived VOC OH reactivity at high time resolution. We co-located multiple MiniPID sensors with a PTR-ToF-MS reference and a thermohygrometer, harmonized the measurements to a common temporal grid, and evaluated a suite of regression algorithms, emphasizing ensemble methods capable of capturing nonlinear effects. To better reflect realistic deployment conditions under temporal autocorrelation, we adopt a time-aware validation strategy in which models are trained on a contiguous short calibration period and evaluated on subsequent “out-of-time” data. The objective is not only improved agreement with the reference metric, but also improved inter-sensor consistency, supporting practical harmonization of PID sensor networks for high-temporal-resolution monitoring of VOC photochemical reactivity.

## 2. Materials and Methods

### 2.1. Instruments and Data Collection

Four PID sensors (MiniPID 2 PPB, ION Science, Royston, UK) were deployed. Each unit uses a 10.6 eV ultraviolet (UV) lamp to ionize gas-phase compounds with ionization potentials below the lamp energy; the resulting ion current is converted to an electrical signal reported by the instrument as an isobutylene-equivalent VOC reading. The MiniPID devices used here integrate the PID module with an active sampling pump, controller, GPS, and wireless telemetry for real-time reporting. Before the co-location campaign, all sensors were factory calibrated with isobutylene and the UV-lamp windows were cleaned following the manufacturer’s guidelines.

A PTR-ToF-MS (Model 2000, IONICON, Innsbruck, Austria) served as the time-resolved reference instrument. PTR-ToF-MS measures many VOCs online by proton-transfer reactions in a drift tube and provides second-level temporal resolution [27]. The instrument is particularly sensitive to many alkenes and aromatics, which are also important contributors to VOC reactivity and are typically within PID sensitivity, although it has limited sensitivity for some compounds (e.g., formaldehyde, propylene, and alkanes). To construct a reference target consistent with the measurable VOC subset, we computed PTR-derived VOC OH reactivity (*R*_OH,PTR_, s^−1^) from a selected compound set (Table S1, Supplementary Material) compiled from 65 target compounds from the TO-15 list, 57 components from the PAMS list, and 13 aldehydes/ketones. *R*_OH,PTR_ was calculated as *R*_OH,PTR_ = Σ_i_ k_OH,i_ [X_i_], where k_OH,i_ is the OH reaction rate constant and [X_i_] is the VOC number density (molecules cm^−3^), yielding units of s^−1^ [25,26]. VOC mixing ratios from PTR-ToF-MS were converted to number densities using the ideal gas law. For isomeric PTR-ToF-MS signals that could not be chromatographically resolved, the average k_OH_ across the isomer group was applied following Wang et al. [28]. It is worth noting *R*_OH,PTR_ here is the VOC-only OH reactivity computed from the selected PTR-ToF-MS-quantified compound set (Table S1), and it is not a direct measurement of total atmospheric OH reactivity, which would also include VOCs not quantified by the PTR-ToF-MS subset and inorganic OH sinks.

In accordance with WMO guidance on low-cost sensor calibration [29], the experimental setup comprised four PID sensors, a thermohygrometer (HC2A-S, Rotronic, Bassersdorf, Switzerland; temperature and relative humidity), and the PTR-ToF-MS co-located in a laboratory with sampling inlets extended outdoors to sample the same ambient air. Data were logged at 3 s resolution (MiniPID response intervals of 3–5 s) and synchronized by timestamp. After quality control, all time series were resampled to 10 s means to establish a common temporal grid while retaining high-frequency variability. The first 24 h of measurements were discarded to reduce warm-up artifacts. Periods with PTR-ToF-MS shutdown, missing data, or obvious anomalies (such as single-sensor spikes identified by cross-sensor comparison) were removed. In total, 131 records (each of 10-s duration) were removed as transient anomalies, and only timestamps with complete records (PID output, temperature, relative humidity, and *R*_OH,PTR_) were retained for model development.

### 2.2. Machine Learning Calibration

Machine-learning calibration was formulated as a supervised regression problem. For each PID unit, a model was trained to predict *R*_OH,PTR_ from the raw PID signal together with temperature and relative humidity. Seven candidate regression algorithms were evaluated: multiple linear regression (MLR), decision tree (DT), gradient boosting (GB), k-nearest neighbors (kNN), random forest (RF), support vector regression (SVR), and extreme gradient boosting (XGBoost), following prior field-calibration studies [30,31,32,33]. Here, linear models are used as an interpretable baseline against which multidimensional machine learning models are compared. The linear regression model is formulated as follows (Equation (1)), in which the reference value (*R*_OH,PTR_) is modeled as a function of the sensor reading (*R*_sensor_), temperature (*T*), and relative humidity (*RH*). All analyses were implemented in Python (version 3.10.11) using the third-party libraries scikit-learn (version 1.5.1) and xgboost (version 2.1.3).

(1) $$R_{OH,PTR}=\beta_{0}+\beta_{1}R_{sensor}+\beta_{2}T+\beta_{3}RH,$$

Model development was conducted in two stages. First, an intra-day random split (85% training, 15% testing) was used to screen algorithms and tune hyperparameters using data from one 24 h period (26 October). The optimized hyperparameters and screening metrics are provided in the Supplementary Material (Tables S2 and S3). Second, to reflect realistic generalization under temporal autocorrelation, a time-aware (“out-of-time”) validation was performed: models were trained on one contiguous 24 h co-location period and evaluated on data from subsequent campaign days. All preprocessing steps were fit using training data only and then applied unchanged to the test period to avoid information leakage.

### 2.3. Metrics for Evaluation

Model performance was quantified using Pearson’s correlation coefficient (*r*), coefficient of determination (R^2^), mean absolute error (MAE), and root mean squared error (RMSE), which are widely used in gas-sensor evaluation and field-calibration studies [6,34,35]. Metrics were computed at the native 10 s resolution; for application-oriented assessment, the calibrated and reference time series were also aggregated to 1 h means before calculating the same metrics.

## 3. Results and Discussion

### 3.1. Environmental Conditions and Observational Data

**Figure 1:**
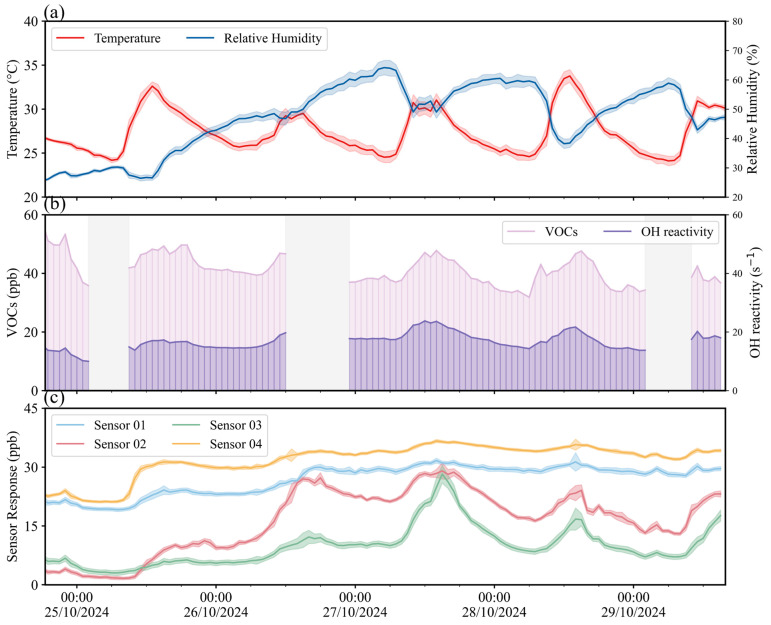
Time series of hourly-averaged raw data: (**a**) temperature and relative humidity, (**b**) PTR-derived summed VOC mixing ratios and PTR-derived VOC OH reactivity, and (**c**) raw PID sensor outputs. The gray box in (**b**) indicates periods of missing data.

Figure 1 summarizes the co-location campaign, showing hourly averages of (a) temperature and relative humidity (RH), (b) PTR-derived reference metrics, and (c) raw PID outputs from the four MiniPID units. During the study period, RH ranged from 24.30% to 69.10% and temperature from 23.10 °C to 35.10 °C, both exhibiting pronounced diurnal variability. A rainfall event late in the campaign increased RH relative to the earlier period (25–26 October). Temperature and RH were generally anti-correlated, with daytime temperature maxima coinciding with RH minima.

PTR-ToF-MS measurements were used to compute two complementary reference quantities from the same selected VOC set (Table S1): the summed VOC mixing ratio (ΣVOC_PTR_, ppb) and the PTR-derived VOC OH reactivity (*R*_OH,PTR_, s^−1^). ΣVOC_PTR_ ranged from 30.23 to 65.57 ppb (mean 41.26 ppb), while *R*_OH,PTR_ ranged from 9.35 to 25.01 s^−1^ (mean 17.06 s^−1^). Although the two metrics showed broadly similar diurnal structure, *R*_OH,PTR_ exhibited smoother variability, consistent with its reactivity-weighted definition. Figure 1c illustrates substantial inter-sensor variability in the raw PID signals: despite identical operating settings and comparable sensor age, the amplitude and short-term fluctuation patterns differed across devices (Sensors 02–03 showed stronger variability than Sensors 01 and 04). These discrepancies underscore the need for sensor-specific calibration and motivate a network harmonization approach.

### 3.2. Evaluation of Calibration Model Performance

As mentioned above, the primary calibration target in this study is PTR-derived VOC *R*_OH,PTR_ because PID sensors are non-speciated and their integrated response depends on VOC composition as well as total abundance. For transparency and to benchmark against a commonly used TVOC surrogate, we also computed the summed VOC mixing ratio from the same VOC set (Table S1). In the present dataset, the association between raw PID output and ΣVOC_PTR_ was consistently weaker than that for *R*_OH,PTR_, consistent with compound-dependent PID response factors and the “many-to-one” calibration challenge. Accordingly, unless otherwise stated, all calibration results below are reported for *R*_OH,PTR_.

Model development proceeded in two stages. First, candidate regression algorithms were screened using an intra-day random split of one calibration day (26 October) to tune hyperparameters and establish an upper bound on achievable performance (Tables S2 and S3). As expected for high-frequency time series, random splits yield optimistic statistics (Pearson’s *r* ≈ 0.93–0.96; Table S2) due to temporal autocorrelation. We therefore focus the discussion on the more realistic out-of-time evaluation, in which models trained on a contiguous 24 h period were applied to the remaining campaign days.

#### 3.2.1. Calibration Accuracy of Machine Learning Models

Out-of-time performance differed across both sensor devices and calibration models. Table 1 summarizes the results for the best-performing approach (XGBoost) at the native 10 s resolution, using the out-of-time split (training on one contiguous 24 h period and evaluation on the remaining campaign days). XGBoost, an efficient gradient-boosting implementation [36] that has been applied successfully in sensor calibration [32,37], achieved strong agreement with *R*_OH,PTR_ across the four PIDs (Pearson’s *r* = 0.72–0.81; R^2^ = 0.50–0.59), with RMSE of 1.92–2.12 s^−1^. These results indicate that a short (24 h) co-location period can yield a transferable calibration for *R*_OH,PTR_ while accommodating substantial inter-sensor differences.

In contrast, the simple linear model fails to capture this complex relationship. Its poor goodness-of-fit is a reflection of this; for example, Sensor 04’s R^2^ was negative (−0.04), while Sensors 01 and 03’s were similarly low, ranging from 0.31 to 0.32. Notably, Sensor 02’s linear model outperformed the XGBoost model in terms of R^2^. This may be attributed to the regularization mechanism in XGBoost, which could excessively penalize the sensor data that already exhibited superior performance. A more straightforward indication of the improvement is observed in the reduction in RMSE. For instance, the RMSE for Device 04 decreased from 3.05 to 2.02 s^−1^.

To assess performance at a temporal scale commonly used for network interpretation and to reduce high-frequency variability, we additionally aggregated both the calibrated predictions and the PTR-derived reference to 1 h means. As shown in Table 2, hourly averaging improved agreement for all four models. For XGBoost, Pearson’s *r* increased to 0.75–0.85 and R^2^ to 0.54–0.64, while RMSE decreased to 1.75–1.97 s^−1^. The improved statistics are expected because averaging reduces random instrumental noise and short-term sampling mismatches between instruments.

**Figure 2:**
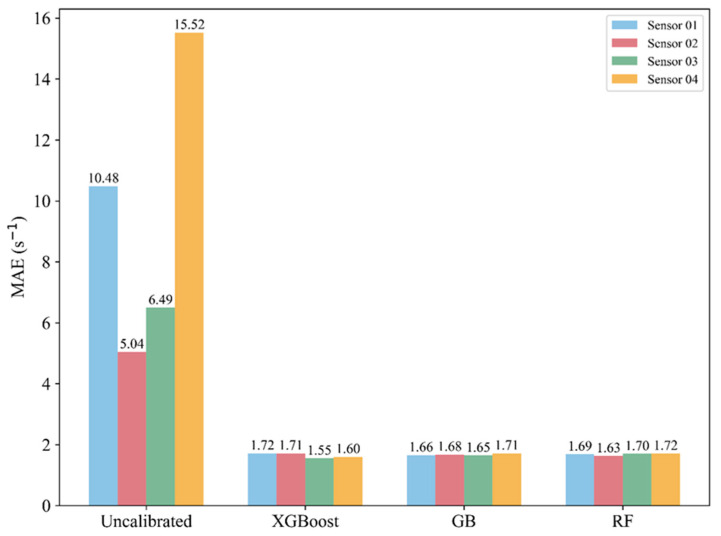
Bar plot of the MAE for four sensors before calibration and after calibration using XGBoost, GB, and RF algorithms, with different colors indicating individual sensors.

To further evaluate calibration performance, we examined the reduction in MAE, which directly reflects the deviation between predicted and reference *R*_OH,PTR_ values [38] (Figure 2). The mean *R*_OH,PTR_ during the campaign was 17.06 s^−1^, whereas the MAE of the raw PID outputs varied widely across sensors (5.04–15.52 s^−1^), demonstrating substantial device-to-device bias prior to calibration. After calibration, MAE decreased markedly for all sensors and models. For Sensors 02 and 03 (better pre-calibration performance), MAE reductions were 66.07–76.04% across GB, RF, and XGBoost. The improvement was even more pronounced for the poorer-performing Sensors 01 and 04, whose post-calibration MAE decreased to ~1.7 s^−1^, corresponding to reductions of 83.64–84.28% and 88.88–89.49%, respectively. Overall, the MAE reductions clustered within a narrow, consistently high range (66.07–89.49%), indicating robust performance despite the many-to-one calibration challenge and large inter-sensor variability.

**Figure 3:**
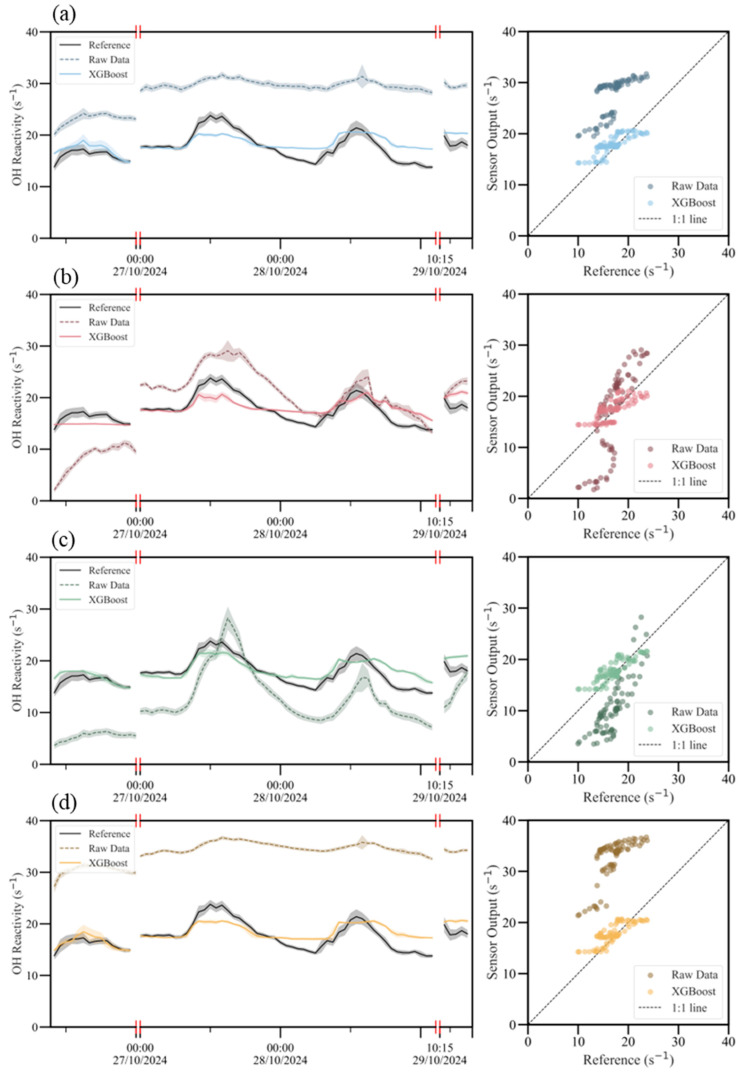
Hourly-averaged time series and scatter plots comparing sensor measurements with reference data before and after calibration for four sensor devices. Panels (**a**–**d**) correspond to Sensor 01–04. Time series are shown as hourly means with error bands.

**Figure 4:**
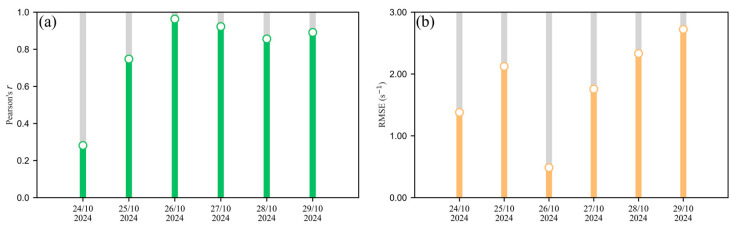
Temporal evolution of model performance for Sensor 01 after calibration with the XGBoost model: (**a**) Pearson’s *r* and (**b**) RMSE (s^−1^). The metrics for October 26 are evaluated on the testing dataset, whereas subsequent metrics are derived from daily prediction data.

Figure 3 illustrates the improvement achieved by the XGBoost calibration for each sensor using hourly means (time series) and 1:1 scatter plots against *R*_OH,PTR_. Before calibration, Sensors 01, 02, and 04 generally overestimated *R*_OH,PTR_, whereas Sensor 03 predominantly underestimated it; additionally, Sensors 01 and 04 showed reduced sensitivity to subtle fluctuations and peak structures in the reference. After calibration, the time series tracked the reference more closely, and the scatter points clustered around the 1:1 line, indicating that systematic bias was largely removed and remaining errors were primarily random. As shown in Figure 4, the out-of-time performance of the Sensor 01 XGBoost calibration degrades as the evaluation period moves farther from the 24-h co-location window. Performance is highest within the calibration day (26 October; Pearson’s *r* > 0.96 for the held-out test segment), whereas applying the same trained model to data outside this window yields systematically larger errors. In particular, RMSE increases progressively over subsequent days, reaching ~2.7 s^−1^ on 29 October (ΔRMSE > 2 s^−1^ between 26 and 29 October), illustrating rapid accumulation of prediction error during deployment. Pearson’s *r* also deteriorates outside the calibration window, with the lowest value (<0.30) observed on 24 October. Collectively, these results indicate limited temporal transferability of a single short co-location calibration under evolving sensor state (e.g., lamp/window effects) and/or changing environmental/emission regimes. For practical deployments, we therefore recommend sub-weekly recalibration (with weekly as an upper bound for this dataset), and more frequent updates (every few days) when high quantitative accuracy is required or when meteorology/VOC-mixture regimes shift. More generally, a hybrid strategy combining scheduled recalibration with performance-triggered checks during periodic co-locations (e.g., recalibrate when *r* falls below, or RMSE exceeds, an application-specific threshold) can help maintain data quality over extended deployments. Feature attribution analysis (Section 3.3 and Figure S1) further indicates that the relative influence of the raw PID signal versus meteorological covariates is sensor-dependent, underscoring the need for sensor-specific models and rigorous data quality control.

#### 3.2.2. Sensor Consistency After Calibration

**Figure 5:**
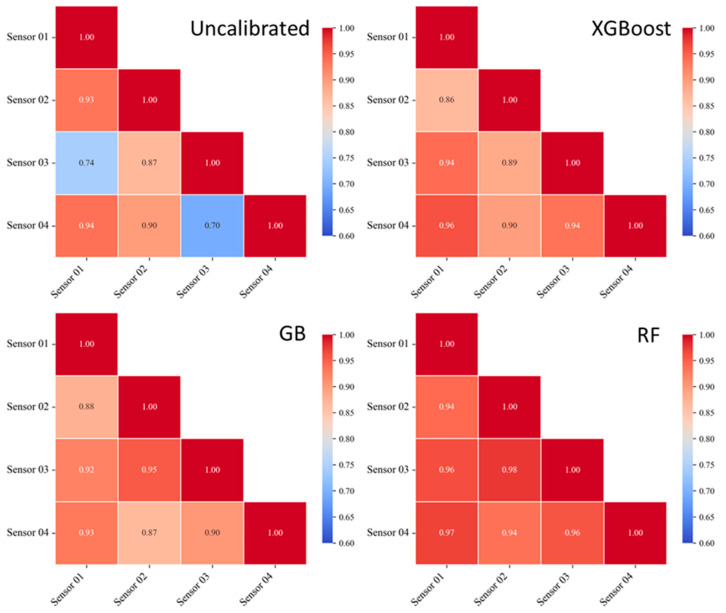
Pairwise correlation heatmaps of sensor measurements before calibration and after calibration using the XGBoost, GB, and RF models.

In addition to comparing with the reference instrument, we also examined the inter-sensor response agreement, the improvement of which also reflects the effectiveness of the calibration models. As shown in Figure 5, the correlation coefficients between sensors were unevenly distributed before calibration. Pairs 01–03 and 03–04 showed weak correlation, consistent with the significantly higher fluctuation amplitude of Sensor 03 compared to Sensors 01 and 04 in Figure 1. After calibration, the lower correlation coefficients increased significantly (e.g., from 0.70 to above 0.90), and the correlations of other pairs also shifted toward higher values. Among the models, RF achieved the most pronounced and uniform improvement, with post-calibration inter-sensor correlations concentrating in the range of 0.94–0.98. The other two models slightly reduced the correlation for a few pairs that were already rather strong, all of which involved Sensors 01 and 02.

**Figure 6:**
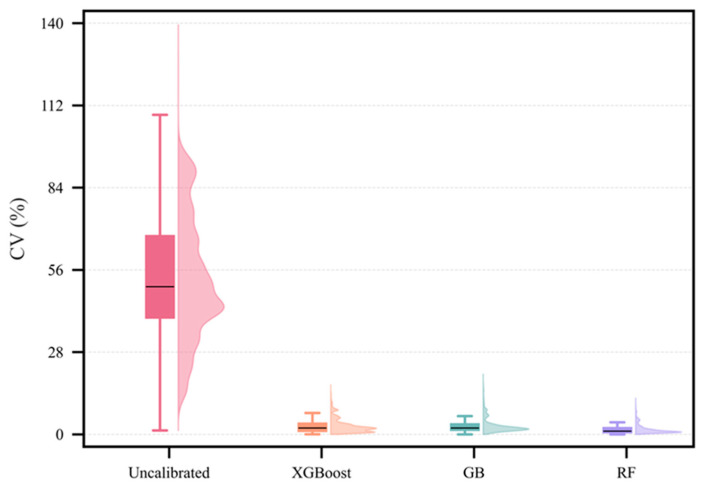
Distribution of the CV among the four sensor devices at each time point before calibration and after calibration using the XGBoost, GB, and RF models, shown as box–violin plots. The box represents the interquartile range, the line inside the box denotes the median, and the whiskers extend to 1.5 times the interquartile range.

The coefficient of variation (CV) was used as a complementary metric to correlation, measuring the dispersion of responses across different devices at the same time point. As presented in Figure 6, the pre-calibration CV was widely distributed with a high median (50.29%), exhibiting a multimodal and long-tailed form. This indicates poor agreement and significant volatility, including instances where CV reached 139.41%. After calibration, CV decreased markedly and its distribution became much tighter. The RF model performed most effectively, yielding a median post-calibration CV of 1.10% and confining the majority of values below 2%. All three models lowered the CV to the 0–21% range by eliminating extreme outliers. To further diagnose sensor-to-sensor agreements, pairwise sensor-sensor regression and Bland–Altman analysis were conducted before and after calibration (Table S5 and Figure S2). These metrics demonstrate that calibration not only increases correlation but also reduces systematic bias and improves 1:1 agreement across devices.

In conclusion, the calibration models not only improved agreement with the reference instrument but also significantly enhanced the internal consistency of the sensor network. Correlation analysis confirmed better-synchronized trends after calibration, while CV analysis demonstrated a substantial reduction in the dispersion of response magnitudes. The deployment of dependable real-time monitoring networks is greatly aided by these findings.

### 3.3. Error Characteristics and SHAP Analysis

**Figure 7:**
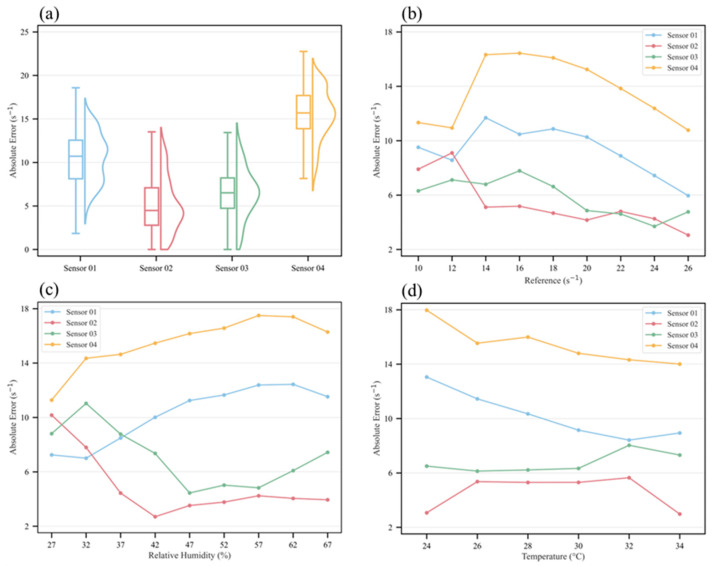
Distribution and environmental dependence of absolute errors for the sensor measurements. Panel (**a**) shows box–violin plots of absolute errors for the four sensor devices, where the box represents the interquartile range, the line inside the box denotes the median, and the whiskers extend to 1.5 times the interquartile range. Panels (**b**–**d**) show the dependence of absolute error on the reference *R*_OH,PTR_ level, relative humidity, and temperature, respectively.

Figure 7 illustrates the error characteristics of the sensors under different conditions. Sensor 03 shows the most symmetric and concentrated error distribution with the narrowest interquartile range. Except for Sensor 03, which exhibits a unimodal distribution, all other sensors display multimodal distributions, suggesting that errors vary under different environmental regimes. Although the distributions of Sensors 01 and 04 are roughly symmetric, they are more dispersed. The box plot of Sensor 02 indicates a low median and an extended upper tail, suggesting that most errors are clustered in the lower range. Compared with Sensors 01 and 04, the errors of Sensors 02 and 03 are primarily concentrated within 2.79–8.22 s^−1^, which is consistently closer to the reference values.

While panel (a) reveals overall inter-sensor differences in error distributions, panels (b), (c), and (d) detail the conditional dependence of absolute errors on the reference *R*_OH,PTR_ level, RH, and temperature, respectively. Panel (b) shows a nonlinear relationship between absolute error and *R*_OH,PTR_. Errors are typically larger and more variable below ~16 s^−1^, likely due to reduced signal-to-noise and nonlinear response at the low end of the range. The influence of temperature is relatively minor within the observed range, generally causing error variations within ~5 s^−1^. In contrast, RH exerts a more systematic effect: errors increase with RH (or decrease and then increase), with the difference between minimum and maximum error reaching up to 7.47 s^−1^. These findings underscore the necessity of including meteorological covariates in calibration models; none of the sensors maintained stable performance across varying environmental conditions.

**Figure 8:**
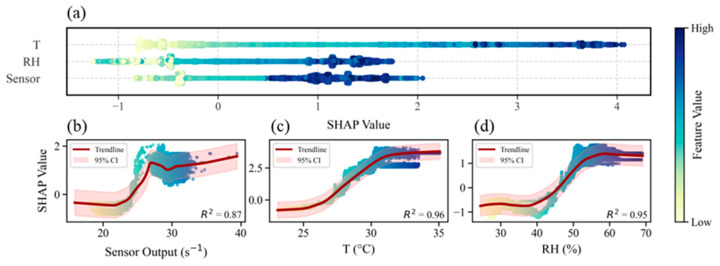
SHAP analysis of the XGBoost model for Sensor 01. Panel (**a**) shows the SHAP summary plot, and panels (**b**–**d**) show SHAP dependence plots for the three input features with LOWESS fits.

SHAP (SHapley Additive exPlanations) analysis, based on Shapley values from coalitional game theory, was applied to interpret the machine-learning models by quantifying the contribution of each input feature [39]. Taking the XGBoost model calibrated for Sensor 01 as an example, Figure 8 visualizes the SHAP contributions of temperature, RH, and the raw sensor output (results for other sensors are provided in the Supplementary Material, Figure S1). For Sensor 01, temperature is the most important feature, followed by RH and then the sensor output. This ranking, however, is device-specific: while Sensor 03 shares the same order as Sensor 01, the sensor output is most influential for Sensor 02; for Sensor 04, temperature remains the dominant feature but is followed by the sensor output.

The dependence plots (Figure 8b–d) illustrate the relationship between SHAP values and individual features using scatter points and locally weighted scatterplot smoothing (LOWESS) curves [40]. For Sensor 01, SHAP values show a negative association with both sensor output and RH at lower values, transitioning to positive contributions as the features increase. For Sensors 02–04, the dependence plots show stable positive trends in the mid-range of feature values but exhibit irregular behavior at the extremes. This segmented pattern is likely attributable to non-uniform data density across the feature space, where turning points correspond to shifts in sampling frequency within specific value ranges.

In summary, SHAP analysis demonstrates that dominant features vary among sensor devices and that the relationships between predictors and *R*_OH,PTR_ are primarily nonlinear. These insights support the use of nonlinear ML models for PID calibration and can guide sensor-specific model optimization by identifying the most influential environmental and instrumental drivers of prediction variability.

## 4. Conclusions

PID sensors enable fast, low-cost monitoring of ambient VOC variability, but their quantitative utility is limited by strong sensitivity to temperature and relative humidity, sensor drift, and substantial inter-sensor variability. In addition, the non-speciated nature of PID measurements creates a “many-to-one” calibration challenge in which the same PID response can be produced by different VOC mixtures. To address these constraints in a practical manner, this study developed and evaluated a short-term machine-learning calibration workflow that maps PID outputs and meteorological covariates to a photochemically relevant reference metric, *R*_OH,PTR_, computed from PTR-ToF-MS measurements and OH reaction rate constants.

Using four co-located MiniPID sensors, a PTR-ToF-MS, and a thermohygrometer, we demonstrated that ensemble learning methods provide robust calibration performance even when trained on a single contiguous 24 h co-location period and evaluated under a time-aware (out-of-time) validation strategy. In out-of-time evaluation at 10 s resolution, the best-performing model (XGBoost) achieved strong agreement with *R*_OH,PTR_ across sensors (Pearson’s *r* = 0.72–0.81; R^2^ = 0.50–0.59; RMSE = 1.92–2.12 s^−1^). When aggregated to 1 h means, agreement improved further (*r* = 0.75–0.85; R^2^ up to 0.64; RMSE = 1.75–1.97 s^−1^), consistent with reduced short-term noise and sampling mismatch. Beyond reference agreement, calibration substantially improved network harmonization: pairwise inter-sensor correlations increased and the dispersion of concurrent sensor responses (coefficient of variation) decreased markedly, indicating that the approach can reduce device-to-device bias and enable more consistent network-level interpretation. In real deployments, the same workflow can be applied in a rolling manner (periodic 24 h re-training on a recent co-location window) to maintain accuracy over longer monitoring periods.

Error diagnostics and SHAP interpretability analysis indicate that the relative influence of meteorological covariates versus raw PID output is sensor-dependent and predominantly nonlinear, supporting the use of nonlinear ML models and reinforcing the need for rigorous data cleaning and sensor-specific calibration. Compared to other representative calibration strategies, such as deep learning–based methods that require large training datasets and operate as black-box models with limited interpretability [41], transfer learning techniques that are not only contingent upon domain similarity but also face fundamental challenges in transferring total OH reactivity/VOC components across different contexts [42], while introducing algorithmic complexity unsuited to low-cost field networks, or multi-sensor fusion approaches that improve accuracy at the expense of increased system cost and scalability [43], the proposed approach offers a data-efficient, interpretable, and low-cost solution for PID network deployment.

Overall, these results show that a short, operationally feasible co-location can yield a transferable calibration for *R*_OH,PTR_, enabling practical deployment of PID networks for high-temporal-resolution VOC reactivity monitoring in urban and industrial settings.

Several limitations and future directions warrant emphasis. First, *R*_OH,PTR_ is derived from the subset of VOCs measurable and selected for the PTR-ToF-MS; thus, it represents VOC-only OH reactivity for the selected compound set, not a direct measurement of total atmospheric OH reactivity. Second, the campaign duration and meteorological range were limited; future studies should test multi-season performance, broader humidity/temperature regimes, and more variable VOC mixture composition. Third, long-term deployments should explicitly address lamp aging and contamination-driven drift by quantifying recalibration frequency requirements and evaluating drift-aware or adaptive calibration strategies. Finally, both training and validation in this study were based on continuous monitoring at the same site, and therefore our results primarily demonstrate short-term transferability under broadly similar mixture types. At the same time, our calibration target (*R*_OH,PTR_) is reactivity-weighted and is dominated by VOC classes (e.g., alkenes and aromatics) that are typically within both PTR-ToF-MS coverage and PID sensitivity, which makes the calibration more chemically meaningful than a simple TVOC sum. Within the campaign, we observed day-to-day variability in the relative contributions of reactive VOC classes, and the model maintained useful performance under these variations, suggesting some robustness to moderate composition changes. However, we recommend a new short co-location when deploying in environments with fundamentally different VOC mixtures (e.g., different industrial solvent profiles or indoor settings), and expanding the framework to multi-site transfer (e.g., domain adaptation or consensus approaches) and incorporating additional covariates (e.g., pressure) would further improve robustness for real-world monitoring networks.

## Figures and Tables

**Table 1:** Evaluation metrics of XGBoost model (10 s time resolution).

Model	Sensor	Pearson’s *r*	R^2^	MAE (s^−1^)	RMSE (s^−1^)
XGBoost	Sensor 01	0.78	0.50	1.72	2.12
	Sensor 02	0.72	0.51	1.71	2.09
	Sensor 03	0.81	0.59	1.55	1.92
	Sensor 04	0.78	0.54	1.60	2.02

**Table 2:** Evaluation metrics of calibration models (1-h time resolution).

Model	Sensor	Pearson’s *r*	R^2^	MAE (s^−1^)	RMSE (s^−1^)
DT	Sensor 01	0.78	0.54	1.55	1.98
DT	Sensor 02	0.77	0.58	1.52	1.89
DT	Sensor 03	0.77	0.53	1.61	1.99
DT	Sensor 04	0.79	0.57	1.54	1.90
GB	Sensor 01	0.85	0.57	1.53	1.92
GB	Sensor 02	0.77	0.57	1.56	1.92
GB	Sensor 03	0.79	0.59	1.54	1.87
GB	Sensor 04	0.79	0.54	1.56	1.97
RF	Sensor 01	0.80	0.54	1.54	1.97
RF	Sensor 02	0.78	0.57	1.52	1.90
RF	Sensor 03	0.77	0.54	1.57	1.98
RF	Sensor 04	0.77	0.53	1.57	2.00
XGBoost	Sensor 01	0.82	0.54	1.59	1.97
XGBoost	Sensor 02	0.75	0.55	1.60	1.94
XGBoost	Sensor 03	0.85	0.64	1.41	1.75
XGBoost	Sensor 04	0.82	0.59	1.46	1.86

## Data Availability

The data presented in this study are available on request from the corresponding author. The data are not publicly available due to internal policy of the university.

## Supplementary Materials

The following supporting information can be downloaded at: https://www.mdpi.com/article/10.3390/s26051428/s1, Table S1: List of selected VOCs used to compute PTR-derived VOC OH reactivity (*R*_OH,PTR_) and summed VOC mixing ratio (ΣVOC_PTR_). Compounds appearing in multiple source lists are listed once; the “Source list(s)” field indicates all lists in which the compound appears; Table S2: Screening performance of candidate calibration models for *R*_OH,PTR_ using an intra-day random split (85%/15%) on the 24-h calibration dataset (26 October). Note that random splits of high-frequency time series can yield optimistic statistics due to temporal autocorrelation; out-of-time performance is reported in Table S4; Table S3: Tuned hyperparameters for each model and sensor (derived from the intra-day random-split screening on 26 October); Table S4: Out-of-time evaluation metrics for *R*_OH,PTR_ at 10-s resolution. Models were trained on one contiguous 24-h co-location period and evaluated on data from subsequent campaign days (out-of-time); Table S5: Slopes and intercepts before and after calibration. “Before” and “after” denote pre-calibration and post-calibration, respectively; Figure S1: SHAP analysis of the XGBoost model predicting *R*_OH,PTR_ for Sensors 01–04. Panels (a–d) correspond to Sensors 01–04, respectively, showing the SHAP summary plot and SHAP dependence plots for sensor output, temperature, and relative humidity with LOWESS fits; Figure S2: Bland-Altman analysis of the XGBoost model before and after calibration for (a) Sensors 01 and 03, (b) Sensors 02 and 03, and (c) Sensors 03 and 04; Figure S3: The workflow diagram to document the complete preprocessing and modeling pipeline.
