# Supplementary material for: Short-Term Machine-Learning Calibration of PID Sensors for Ambient VOC OH Reactivity
Source: Sensors (Basel). 2026 Feb 25;26(5):1428. doi: 10.3390/s26051428 (PMC12987072; doi:10.3390/s26051428)
Supplement: Supplementary file 1 [file sensors-26-01428-s001.zip › sensors-4129575-supplementary.pdf]

Supplementary Information

Short-Term Machine-Learning Calibration of PID Sensors for Ambient VOC OH reactivity

Han Yang [1,2], Wei Song [1,*], Xiaoyang Wang [1], Jianlin Cheng [1], Chenglei Pei [3], Duohong Chen [4], Zhuoyue Ren [1,2], Xinyi Li [1,2], Xiangyu Zhang [1,2], Xiaodie Pang [1,2], Xue Yu [1,2], Jianqiang Zeng [1], Yanli Zhang [1], and Xinming Wang [1,2,*]

[1] State Key Laboratory of Advanced Environmental Technology and Guangdong Key Laboratory of Environmental Protection and Resources Utilization, Guangzhou Institute of Geochemistry, Chinese Academy of Sciences, Guangzhou 510640, China
[2] College of Resources and Environment, University of Chinese Academy of Sciences, Beijing 100049, China
[3] Guangzhou Sub-branch of Guangdong Ecological and Environmental Monitoring Center, Guangzhou 510006, China
[4] Guangdong Ecological Environment Monitoring Center, Environmental Key Laboratory of Regional Air Quality Monitoring, Ministry of Ecology and Environment, Guangzhou 510308, China
*Corresponding author: songwei@gig.ac.cn (W.S.); wangxm@gig.ac.cn (X.W.)

This document provides supplementary tables and figures supporting the manuscript "Short-Term Machine-Learning Calibration of PID Sensors for Ambient VOC OH Reactivity". The primary calibration target is PTR-derived VOC OH reactivity ($R_{OH,PTR}$, s^{-1}), computed from a selected VOC set measured by PTR-ToF-MS using published OH reaction rate constants. For transparency, the summed VOC mixing ratio (ΣVOC_{PTR}, ppb) based on the same VOC set is also defined as an auxiliary reference metric.

S1. Selected VOC set used to compute $R_{OH,PTR}$ and ΣVOC_{PTR}

Table S1. List of selected VOCs used to compute PTR-derived VOC OH reactivity ($R_{OH,PTR}$) and summed VOC mixing ratio (ΣVOC_{PTR}). Compounds appearing in multiple source lists are listed once; the "Source list(s)" field indicates all lists in which the compound appears.

Compound name	Formula	Source list(s)
1,1,1-Trichloroethane	C2H3Cl3	TO-15
1,1,2-Trichloroethane	C2H3Cl3	TO-15
1,1-Dichloroethane	C2H4Cl2	TO-15
1,2,3-Trimethylbenzene	C9H12	TO-15
1,2,4-Trimethylbenzene	C9H12	TO-15
1,2-Dibromoethane	C2H4Br2	TO-15
1,2-Dichlorobenzene	C6H4Cl2	TO-15
1,2-Dichloroethane	C2H4Cl2	TO-15
1,2-Dichloropropane	C3H6Cl2	TO-15
1,3,5-Trimethylbenzene	C9H12	TO-15
1,3-Butadiene	C4H6	PAMS; TO-15
1,3-Dichlorobenzene	C6H4Cl2	TO-15
1,3-Dichloropropane	C3H6Cl2	TO-15
1,4-Dichlorobenzene	C6H4Cl2	TO-15
1,4-Dioxane	C4H8O2	TO-15
1-Butene	C4H8	PAMS
2-Methylpentane	C6H14	PAMS
3-Methylpentane	C6H14	PAMS
Acetaldehyde	C2H4O	Aldehydes/Ketones; PAMS
Acetone	C3H6O	Aldehydes/Ketones; PAMS
Acetophenone	C8H8O	Aldehydes/Ketones
Acetylene	C2H2	PAMS
Acrylonitrile	C3H3N	TO-15
Benzaldehyde	C7H6O	Aldehydes/Ketones; PAMS
Benzene	C6H6	PAMS; TO-15
Benzyl chloride	C7H7Cl	TO-15
Bromomethane	CH3Br	TO-15
Butylbenzene	C10H14	PAMS
Carbon disulfide	CS2	TO-15
Carbon tetrachloride	CCl4	TO-15
Chlorobenzene	C6H5Cl	TO-15
Chloroform	CHCl3	TO-15
Chloromethane	CH3Cl	TO-15
Cyclohexane	C6H12	PAMS
Cyclohexanone	C6H10O	Aldehydes/Ketones
Cyclopentane	C5H10	PAMS
Decane	C10H22	TO-15
Dichloromethane	CH2Cl2	TO-15
Epichlorohydrin	C3H5ClO	TO-15
Ethane	C2H6	PAMS
Ethene	C2H4	PAMS
Ethyl tert-butyl ether (ETBE)	C6H14O	TO-15
Ethylbenzene	C8H10	PAMS; TO-15
Ethylcyclohexane	C8H16	PAMS
Formaldehyde	CH2O	Aldehydes/Ketones
Heptane	C7H16	TO-15

Hexane	C6H14	TO-15
Isobutane	C4H10	PAMS
Isobutene	C4H8	PAMS
Isopentane	C5H12	PAMS
Isoprene	C5H8	PAMS; TO-15
Isopropylbenzene	C9H12	TO-15
Isopropylbenzene (Cumene)	C9H12	TO-15
MEK (2-Butanone)	C4H8O	Aldehydes/Ketones; PAMS
Methacrolein	C4H6O	Aldehydes/Ketones; PAMS
Methyl isobutyl ketone	C6H12O	Aldehydes/Ketones
Methyl tert-butyl ether (MTBE)	C5H12O	TO-15
Methyl vinyl ketone	C4H6O	Aldehydes/Ketones; PAMS
Methylcyclohexane	C7H14	PAMS
Methylcyclopentane	C6H12	PAMS
MTBE	C5H12O	PAMS
n-Butane	C4H10	PAMS
n-Butylbenzene	C10H14	TO-15
n-Butyraldehyde	C4H8O	Aldehydes/Ketones; PAMS
n-Decane	C10H22	PAMS
n-Dodecane	C12H26	PAMS
n-Heptane	C7H16	PAMS
n-Hexadecane	C16H34	PAMS; TO-15
n-Hexane	C6H14	PAMS
n-Nonane	C9H20	PAMS
n-Octane	C8H18	PAMS
n-Pentadecane	C15H32	PAMS
n-Pentane	C5H12	PAMS
n-Propylbenzene	C9H12	TO-15
n-Tetradecane	C14H30	PAMS
n-Tridecane	C13H28	PAMS
n-Undecane	C11H24	PAMS
n-Valeraldehyde	C5H10O	Aldehydes/Ketones
Nonane	C9H20	TO-15
Octane	C8H18	TO-15
Propane	C3H8	PAMS
Propene	C3H6	PAMS
Propionaldehyde	C3H6O	Aldehydes/Ketones; PAMS
Propylbenzene	C9H12	PAMS
sec-Butylbenzene	C10H14	TO-15
Styrene	C8H8	PAMS; TO-15
tert-Amyl methyl ether (TAME)	C6H14O	TO-15
tert-Butylbenzene	C10H14	TO-15
Tetrachloroethylene	C2Cl4	TO-15
Toluene	C7H8	PAMS; TO-15
Trichloroethylene	C2HCl3	TO-15
Trimethylbenzenes	C9H12	PAMS
Vinyl acetate	C4H6O2	TO-15
Vinyl bromide	C2H3Br	TO-15
Vinyl chloride	C2H3Cl	TO-15
Xylenes (m,p,o)	C8H10	PAMS; TO-15

S2. Model screening (intra-day random split) and tuned hyperparameters

Table S2. Screening performance of candidate calibration models for $R_{OH,PTR}$ using an intra-day random split (85%/15%) on the 24-h calibration dataset (26 October). Note that random splits of high-frequency time series can yield optimistic statistics due to temporal autocorrelation; out-of-time performance is reported in Table S4.

Model	Sensor	Pearson's r	R^2	MAE (s^{-1})	RMSE (s^{-1})
DT	Sensor 01	0.95	0.91	0.40	0.55
DT	Sensor 02	0.93	0.87	0.40	0.70
DT	Sensor 03	0.94	0.89	0.45	0.62
DT	Sensor 04	0.96	0.91	0.40	0.53
GB	Sensor 01	0.96	0.93	0.37	0.49
GB	Sensor 02	0.94	0.88	0.39	0.68
GB	Sensor 03	0.96	0.91	0.40	0.55
GB	Sensor 04	0.96	0.92	0.38	0.50
RF	Sensor 01	0.96	0.93	0.37	0.48
RF	Sensor 02	0.94	0.88	0.38	0.67
RF	Sensor 03	0.96	0.92	0.40	0.53
RF	Sensor 04	0.96	0.93	0.37	0.48
XGBoost	Sensor 01	0.96	0.93	0.37	0.49
XGBoost	Sensor 02	0.94	0.88	0.38	0.67
XGBoost	Sensor 03	0.96	0.92	0.40	0.53
XGBoost	Sensor 04	0.96	0.92	0.38	0.49

Table S3. Tuned hyperparameters for each model and sensor (derived from the intra-day random-split screening on 26 October).

Model	Sensor	Hyperparameter	Value
DT	Sensor 01	max_depth	8
DT	Sensor 01	min_samples_leaf	8
DT	Sensor 01	min_samples_split	2
DT	Sensor 02	max_depth	5
DT	Sensor 02	min_samples_leaf	10
DT	Sensor 02	min_samples_split	3
DT	Sensor 03	max_depth	8
DT	Sensor 03	min_samples_leaf	6
DT	Sensor 03	min_samples_split	2
DT	Sensor 04	max_depth	8
DT	Sensor 04	min_samples_leaf	10
DT	Sensor 04	min_samples_split	7
GB	Sensor 01	learning_rate	0.032
GB	Sensor 01	max_depth	4
GB	Sensor 01	n_estimators	189
GB	Sensor 01	subsample	0.538
GB	Sensor 02	learning_rate	0.083
GB	Sensor 02	max_depth	5
GB	Sensor 02	n_estimators	51
GB	Sensor 02	subsample	0.886
GB	Sensor 03	learning_rate	0.025
GB	Sensor 03	max_depth	6
GB	Sensor 03	n_estimators	200
GB	Sensor 03	subsample	0.519
GB	Sensor 04	learning_rate	0.067
GB	Sensor 04	max_depth	5
GB	Sensor 04	n_estimators	57
GB	Sensor 04	subsample	0.552
RF	Sensor 01	max_depth	13
RF	Sensor 01	max_features	0.300
RF	Sensor 01	min_samples_leaf	1
RF	Sensor 01	min_samples_split	2
RF	Sensor 01	n_estimators	500
RF	Sensor 02	max_depth	10
RF	Sensor 02	max_features	0.302
RF	Sensor 02	min_samples_leaf	1
RF	Sensor 02	min_samples_split	4
RF	Sensor 02	n_estimators	265
RF	Sensor 03	max_depth	12
RF	Sensor 03	max_features	0.300
RF	Sensor 03	min_samples_leaf	1
RF	Sensor 03	min_samples_split	2
RF	Sensor 03	n_estimators	100
RF	Sensor 04	max_depth	10
RF	Sensor 04	max_features	0.302
RF	Sensor 04	min_samples_leaf	1
RF	Sensor 04	min_samples_split	10
RF	Sensor 04	n_estimators	165
XGBoost	Sensor 01	colsample_bytree	1

XGBoost	Sensor 01	learning_rate	0.049
XGBoost	Sensor 01	max_depth	4
XGBoost	Sensor 01	n_estimators	200
XGBoost	Sensor 01	subsample	0.500
XGBoost	Sensor 02	colsample_bytree	0.900
XGBoost	Sensor 02	learning_rate	0.044
XGBoost	Sensor 02	max_depth	6
XGBoost	Sensor 02	n_estimators	157
XGBoost	Sensor 02	subsample	0.952
XGBoost	Sensor 03	colsample_bytree	1
XGBoost	Sensor 03	learning_rate	0.152
XGBoost	Sensor 03	max_depth	2
XGBoost	Sensor 03	n_estimators	176
XGBoost	Sensor 03	subsample	0.735
XGBoost	Sensor 04	colsample_bytree	1
XGBoost	Sensor 04	learning_rate	0.3
XGBoost	Sensor 04	max_depth	2
XGBoost	Sensor 04	n_estimators	100
XGBoost	Sensor 04	subsample	1

S3. Out-of-time evaluation (time-aware validation)

Table S4. Out-of-time evaluation metrics for $R_{OH,PTR}$ at 10-s resolution. Models were trained on one contiguous 24-h co-location period and evaluated on data from subsequent campaign days (out-of-time).

**Model**	**Sensor**	**Pearson's r**	**R²**	**MAE (s⁻¹)**	**RMSE (s⁻¹)**
DT	Sensor 01	0.73	0.46	1.77	2.19
DT	Sensor 02	0.74	0.53	1.65	2.04
DT	Sensor 03	0.72	0.47	1.76	2.17
DT	Sensor 04	0.73	0.49	1.74	2.13
GB	Sensor 01	0.81	0.52	1.66	2.06
GB	Sensor 02	0.74	0.52	1.68	2.06
GB	Sensor 03	0.75	0.54	1.65	2.02
GB	Sensor 04	0.74	0.49	1.71	2.14
RF	Sensor 01	0.76	0.50	1.69	2.11
RF	Sensor 02	0.75	0.53	1.63	2.04
RF	Sensor 03	0.73	0.49	1.70	2.13
RF	Sensor 04	0.73	0.48	1.72	2.14
XGBoost	Sensor 01	0.78	0.50	1.72	2.12
XGBoost	Sensor 02	0.72	0.51	1.71	2.09
XGBoost	Sensor 03	0.81	0.59	1.55	1.92
XGBoost	Sensor 04	0.78	0.54	1.60	2.02

S4. Sensor-to-sensor agreement

Table S5. Slopes and intercepts before and after calibration. “Before” and “after” denote pre-calibration and post-calibration, respectively.

Sensors	Model	Slope (before)	Intercept (before)	Slope (after)	Intercept (after)
01 vs. 02	XGBoost	1.94	-36.60	0.92	0.81
01 vs. 03	XGBoost	1.04	-17.90	1.06	-1.35
01 vs. 04	XGBoost	1.10	2.15	1.02	-0.44
02 vs. 03	XGBoost	0.58	0.95	0.94	1.39
02 vs. 04	XGBoost	0.51	23.80	0.89	2.31
03 vs. 04	XGBoost	0.58	25.88	0.88	2.24

S5. Model interpretability

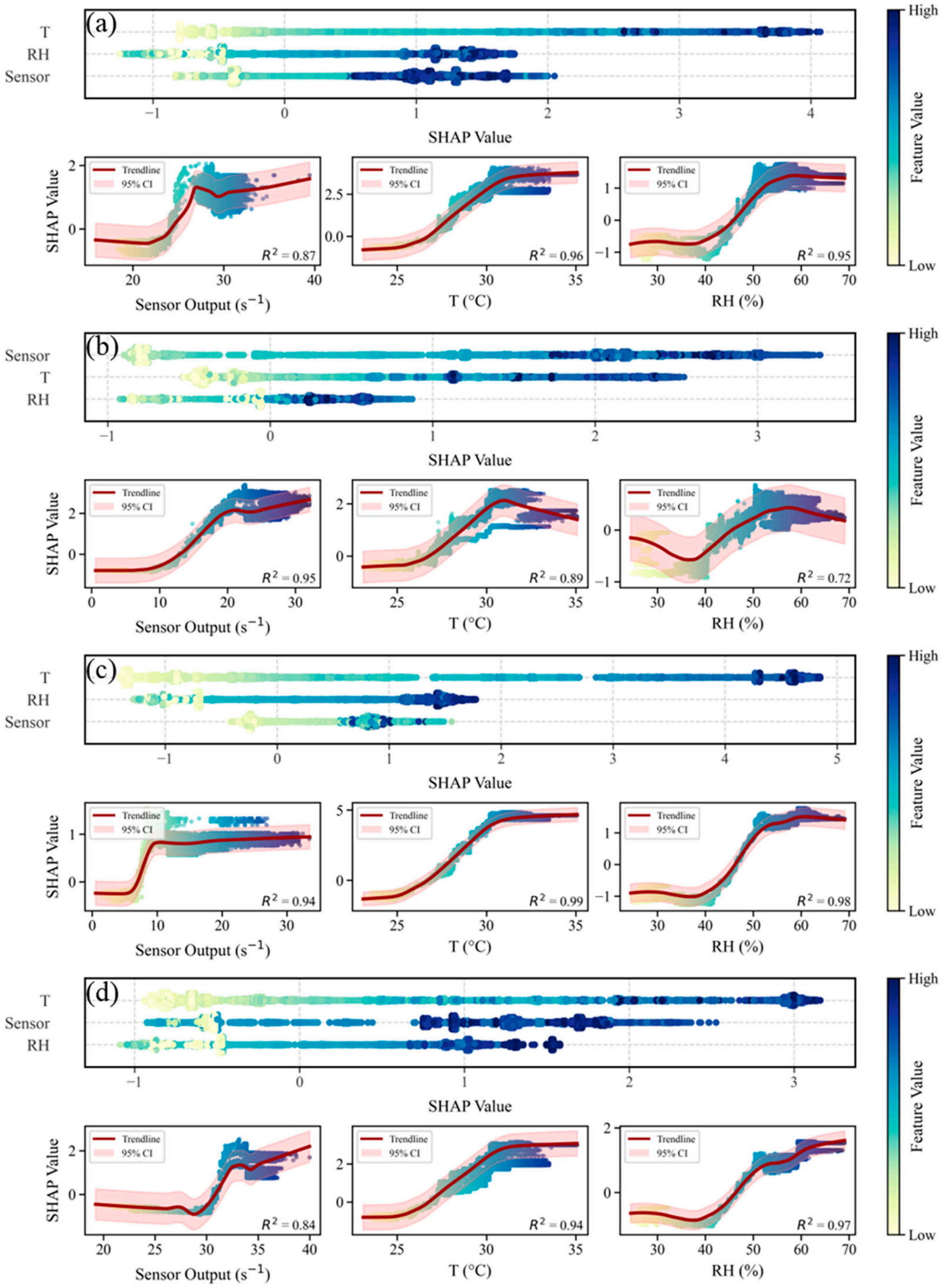

Figure S1. SHAP analysis of the XGBoost model predicting $R_{OH,PTR}$ for Sensors 01–04. Panels (a–d) correspond to Sensors 01–04, respectively, showing the SHAP summary plot and SHAP dependence plots for sensor output, temperature, and relative humidity with LOWESS fits.

S6. Sensor-to-sensor agreement

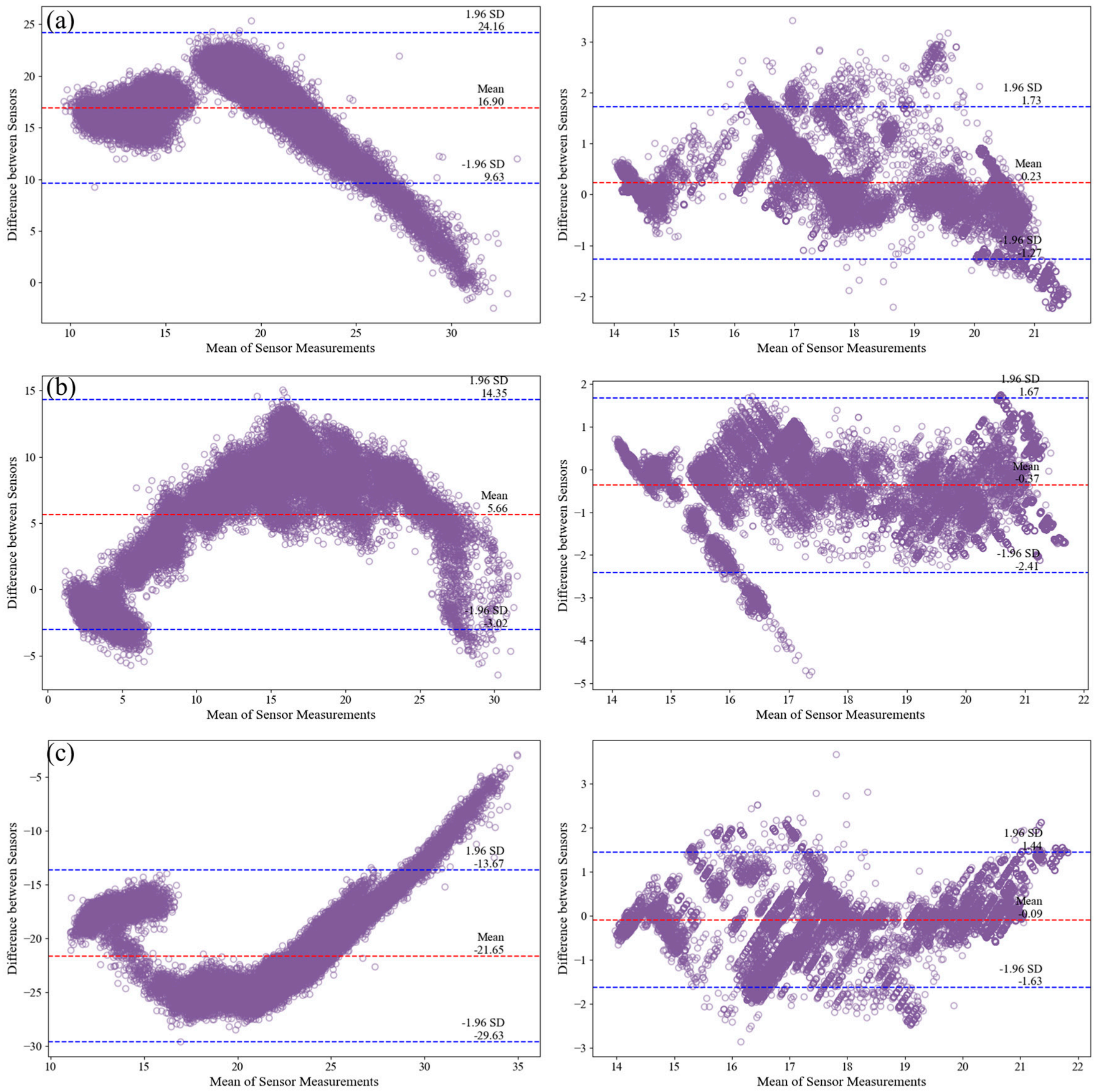

Figure S2. Bland-Altman analysis of the XGBoost model before and after calibration for (a) Sensors 01 and 03, (b) Sensors 02 and 03, and (c) Sensors 03 and 04.

S7. ML processing workflow

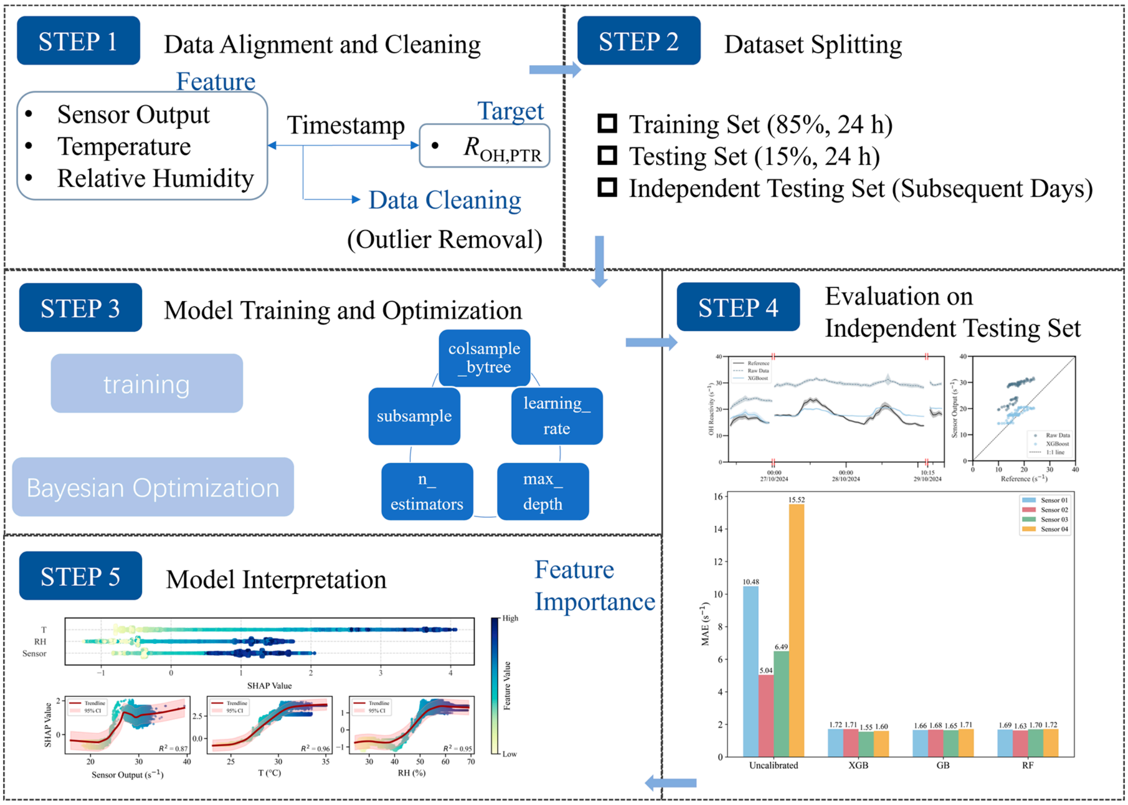

Figure S3. The workflow diagram to document the complete preprocessing and modeling pipeline.
